# Consequences of Obesity on Short-Term Outcomes in Patients Who Underwent Off-Pump Coronary Artery Bypass Grafting Surgery

**DOI:** 10.3390/jcm12051929

**Published:** 2023-03-01

**Authors:** Ihor Krasivskyi, Ilija Djordjevic, Borko Ivanov, Kaveh Eghbalzadeh, Clara Großmann, Stefan Reichert, Medhat Radwan, Rodrigo Sandoval Boburg, Anton Sabashnikov, Christian Schlensak, Thorsten Wahlers, Christian Jörg Rustenbach

**Affiliations:** 1 Department of Cardiothoracic Surgery, University Hospital Cologne, 50937 Cologne, Germany; 2 Department of Cardiothoracic Surgery, Helios Hospital Siegburg, 53721 Siegburg, Germany; 3 Department of Cardiothoracic Surgery, University Hospital Tuebingen, 72076 Tuebingen, Germany

**Keywords:** obesity, BMI, OPCAB, mortality

## Abstract

The correlation between off-pump coronary artery bypass (OPCAB) surgery and obesity-related outcomes is still uncertain. The aim of our study was to analyse the pre-, intra-, and postoperative short-term outcomes between obese and non-obese patients after off-pump bypass surgery. We performed a retrospective analysis from January 2017 until November 2022, including a total of 332 (non-obese (*n* = 193) and obese (*n* = 139)) patients who underwent an OPCAB procedure due to coronary artery disease (CAD). The primary outcome was all-cause in-hospital mortality. Our results showed no difference regarding mean age of the study population between both groups. The use of the T-graft technique was significantly higher (*p* = 0.045) in the non-obese group compared to the obese group. The dialysis rate was significantly lower in non-obese patients (*p* = 0.019). In contrast, the wound infection rate was significantly higher (*p* = 0.014) in the non-obese group compared to the obese group. The all-cause in-hospital mortality rate did not differ significantly (*p* = 0.651) between the two groups. Furthermore, ST-elevation myocardial infarction (STEMI) and reoperation were relevant predictors for in-hospital mortality. Therefore, OPCAB surgery remains a safe procedure even in obese patients.

## 1. Introduction

The prevalence of obesity in Europe has increased dramatically in recent years [1,2,3]. High-grade obesity is associated with comorbidities such as arterial hypertension, dyslipidaemia, and Type 2 diabetes mellitus [4]. Furthermore, research groups have found a correlation between obesity and a higher rate of postoperative complications after a coronary artery bypass grafting (CABG) procedure [5,6]. In contrast, other authors showed no differences regarding the short-term outcomes between obese and non-obese patients after open-heart surgery [7]. Thus, the effect of BMI on postoperative complications after a bypass procedure remains unclear [5,8].

Reeves et al. [8] showed no significant differences in outcomes between obese and non-obese groups after a CABG procedure. However, Simopoulos et al. found a significantly higher prevalence of superficial and deep sternal wound infection rates in obese patients after bypass surgery [5]. Additionally, the mortality rate was significantly lower in the non-obese group compared to the obese one [5]. To date, most studies have compared the relation between a high-grade body mass index (BMI) and on-pump (CABG) procedure complications; only a minority of these studies have investigated the association between obesity and off-pump coronary artery bypass (OPCAB) surgery [9,10,11].

The OPCAB procedure is a crucial treatment for patients with triple vessel disease [10,11]. Particularly for high-risk patients, OPCAB surgery provides the maximum level of safety and avoids complications that may be caused by cardiopulmonary bypass (CPB), such as hepatic, renal, and cardiac damage [12,13]. Moreover, the off-pump strategy may prevent myocardial ischemia [14]. The association between OPCAB surgery and obesity-related outcomes remains uncertain [12,13,14].

Our main objective was to analyse the pre-, intra-, and postoperative short-term outcomes between obese and non-obese patients after OPCAB surgery. Our secondary objective was to identify relevant predictors for in-hospital mortality.

## 2. Materials and Methods

**Figure 1:**
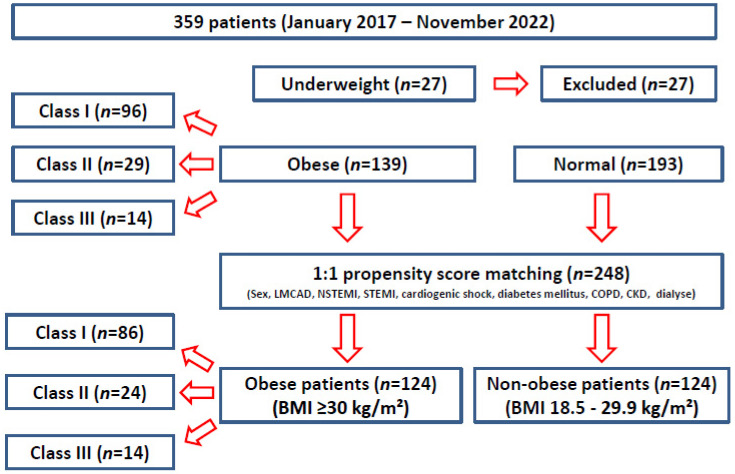
Patient selection and group distribution according to BMI classes.

Retrospective double centre analysis of the OPCAB cohort was performed. From January 2017 until November 2022, a total of 359 patients underwent off-pump coronary artery bypass procedures due to coronary artery disease in the department of cardiovascular surgery in the universities of Cologne and Tuebingen in Germany. To compare the unequal patient groups, a propensity score-based matching (PSM) analysis was applied (Figure 1).

### 2.1. Definition of Obesity

Our methods were previously described in [15]. Based on the World Health Organization (WHO)’s obesity classification, our sample was divided into 6 categories:

- Underweight: BMI <18.5 kg/m^2^
- Normal weight: BMI 18.5–24.9 kg/m^2^
- Overweight: BMI 25.0–29.9 kg/m^2^
- Obese class I: BMI 30.0–34.9 kg/m^2^
- Obese class II: BMI 35.0–39.9 kg/m^2^
- Obese class III: BMI >40.0 kg/m^2^

For analysis of the obesity-dependent factors on clinical outcomes, patients were divided into non-obese (BMI < 30 kg/m^2^, *n* = 193) and obese (BMI ≥ 30 kg/m^2^, *n* = 139) before PSM, and into non-obese (BMI < 30 kg/m^2^, *n* = 124) and obese (BMI ≥ 30 kg/m^2^, *n* = 124) groups after PSM. Underweight patients (*n* = 27) were excluded. In order to provide more specific results through our analysis, we created 3 obesity classes in our sample. Both primary and secondary outcomes were evaluated for patients suffering obese classes I, II, and III.

### 2.2. Surgical Procedure

All patients included in this study underwent OPCAB surgery. Patients who underwent CABG with CPB were excluded. All operations were performed through a median sternotomy. After local stabilisation was achieved with an automatic pod spread for effective visualisation of the anastomotic site, a longitudinal incision of the coronary artery was performed. Subsequently, a temporary shunt was inserted into the lumen of the targeted vessel to allow continuous blood flow during anastomosis and to limit possible bleeding. Monofilament sutures (8–0) were used in most cases. Our methods were previously described in [15].

### 2.3. Data Collection

We collected the data during the patients’ in-hospital stay from the databases of both hospitals. The collected data include the following:

- patients’ baseline characteristics (age, gender, Euroscore II, ejection fraction, left main coronary artery disease, history of Non-ST-elevation myocardial infarction (NSTEMI), history of ST-elevation myocardial infarction (STEMI), cardiogenic shock, previous stenting, previous stroke, reoperation rate, diabetes mellitus, hyperlipidaemia, peripheral vascular disease, arterial hypertension, pulmonary hypertension, chronic obstructive pulmonary disease, chronic kidney disease and dialysis);
- intraoperative characteristics (use of both internal thoracic arteries, total arterial revascularization, T-graft technique, endoscopic saphenous vein harvesting, heartstring use, catecholamine use, temporary pacer use, duration of bypass surgery, extracorporeal membrane oxygenation (ECMO) use, and intra-aortic balloon pump (IABP) use);
- postoperative data (transient ischemic attack (TIA), stroke, delirium, low cardiac output syndrome (LCOS), CK, CK-MB, lactate, creatinine, acute kidney injury, dialysis, wound infection, plastic covering, permanent pacemaker implantation (PPI), bleeding with reoperation, intensive care unit (ICU) stay, hospital stay, and in-hospital mortality);
- primary and secondary endpoints due to obesity classes I, II, and III;
- combined risk factors of in-hospital mortality (age, body mass index, diabetes mellitus, STEMI, NSTEMI, and reoperation rate).

### 2.4. Outcome Analysis

The primary endpoint in our study was all-cause in-hospital mortality after OPCAB surgery. Secondary endpoints were dialysis, bleeding with reoperation, wound infection, and length of in-hospital stay. Furthermore, risk factors for in-hospital mortality were analysed and are presented in our study.

### 2.5. Ethics

This study was realised in accordance with the Declaration of Helsinki (revised version of 2013). The Ethics Committee of the Medical Faculty of the University of Cologne and the Ethics Committee of the Medical Faculty of the University of Tuebingen stated that we are exempted from applying for ethical approval under German law. Purely retrospective clinical studies do not require ethical approval by the ethics committee.

### 2.6. Statistical Methods

All data are presented as continuous or categorical variables. Categorical data are expressed as total numbers and percentages. Continuous data were evaluated for normality using a one-sample Kolmogorov–Smirnov test and were expressed as the mean ± standard deviation (SD) in cases of normally distributed data or as the median (min/max) in cases of non-normally distributed data. Either Pearson’s χ² test or Fisher’s exact test was used for comparison of categorical data, depending on the minimum expected count in each cross-table. Univariate and multivariate analyses were performed using binary logistical regression. Logistical regression was conducted in order to create the predicted variables. A rigorous 1:1 nearest neighbour-matching algorithm without replacement was used with a 0.2 calliper set. *p*-values < 0.05 were considered statistically significant. Statistical analysis was performed using Statistical Package for Social Sciences, version 28.1 (SPSS Inc., Chicago, IL, USA).

## 3. Results

### 3.1. Preoperative Data

Preoperative characteristics of the two groups before (non-obese, *n* = 193; obese, *n* = 139) and after (non-obese, *n* = 124; obese, *n* = 124) PSM are shown in Table 1. Women were significantly more prevalent (*p* = 0.002) in the obese group compared to the non-obese group before PSM. Cardiogenic shock (*p* = 0.033), diabetes mellitus (*p* < 0.001), chronic obstructive lung disease (COPD) (*p* = 0.002), chronic renal insufficiency (*p* = 0.007), and dialysis (*p* = 0.006) were significantly higher in obese patients compared to non-obese patients before PSM. In contrast, left main coronary artery disease (CAD) (*p* = 0.002) was significantly lower in the obese group before PSM. However, preoperative data was well-equalized between the two groups after 1:1 PSM.

### 3.2. Intraoperative Characteristics

The intraoperative data of both groups before (non-obese (*n* = 193) and obese (*n* = 139)) and after (non-obese (*n* = 124) and obese (*n* = 124)) PSM are shown in Table 2. Total arterial revascularization (TAR) (*p* = 0.005) and the use of the T-graft technique (*p* < 0.001) were significantly higher in non-obese patients compared to obese patients before PSM. Likewise, the use of the T-graft technique was significantly higher (*p* = 0.045) in the non-obese group compared to the obese group after PSM. Further intraoperative data did not differ significantly between the two groups.

### 3.3. Postoperative Data

Postoperative data before (non-obese, *n* = 193; obese, *n* = 139) and after (non-obese, *n* = 124; obese, *n* = 124) PSM are summarized in Table 3. The dialysis rate was significantly higher before (*p* < 0.001) and after (*p* = 0.019) PSM in the obese group compared to the non-obese group. In contrast, the wound infection rate was significantly higher (*p* = 0.014) in non-obese patients compared to obese patients. Regarding further secondary outcomes (bleeding requiring reoperation (*p* = 0.216), transient ischemic attack (*p* = 0.102), postoperative delirium (*p* = 0.450), and length of in-hospital stay (*p* = 0.058)), no significant differences were found between the non-obese and obese groups after PSM. The in-hospital mortality rate also did not differ between the two groups (*p* = 0.651) after matching.

### 3.4. Postoperative Data in Obese Patients

Table 4 shows the postoperative data in obese patients, which are separated according to obesity class. Transient ischemic attack was significantly higher (*p* = 0.008) in the obesity class III compared to the other two obesity classes. In contrast, dialysis was significantly higher (*p* < 0.001) in obesity class II compared to the other classes. In-hospital mortality did not differ significantly (*p* = 0.230) between these patients.

### 3.5. Combined Risk Factors of In-Hospital Mortality

The combined risk factors for in-hospital mortality after OPCAB surgery are shown in Table 5. Univariate analysis followed by multivariate analysis showed STEMI and the reoperation rate were relevant predictors for in-hospital mortality. Age, body mass index (BMI), diabetes mellitus, and non-ST-elevation myocardial infarction (NSTEMI) had no relevant impact on mortality.

## 4. Discussion

This study compares non-obese and obese patients’ short-term outcomes after isolated OPCAB surgery. Our study showed that being obese (BMI≥30 kg/m^2^) was not associated with in-hospital mortality (*p* = 0.651) in OPCAB-treated patients. Other secondary outcomes, except dialysis and wound infections, were comparable between the two groups mentioned above. These results are consistent with data in the literature [10,12,13,14], but several authors investigated the short- and long-term results after mixed on-pump and off-pump CABG surgery [16,17]. So far, the data after isolated OPCAB surgery is insufficient [10,12,18].

The impact of obesity on short-term and long-term outcomes after OPCAB surgery still remains controversial [19,20]. Furthermore, authors reported a significantly higher 30-day mortality rate after OPCAB surgery in patients with decreased BMI [10,20]. Potapov et al. [21] analysed a large cohort of patients with low and high BMI. Authors found significantly higher morbidity and mortality rates in the underweight group compared to patients with high BMI [21]. In addition, Engelman et al. [7] mentioned that low BMI was associated with poor outcomes after off-pump cardiac surgery. However, underweight status was not a predictor for the increased mortality rate [7]. Moreover, Straten et al. [18] showed that both underweight status and morbid obesity could be predictors for early mortality after a CABG procedure. Further study showed that very low and very high BMI are associated with worse outcomes after bypass surgery [22]. In contrast, other authors did not show an increased mortality rate in obese patients after a CABG procedure [23]. Prapas et al. [24] analysed the effect of obesity on morbidity and mortality rates after isolated OPCAB procedures. Likewise, authors could not find any significant differences between the obese and non-obese groups [24].

Prabhakar et al. [25] showed that extreme obesity could be a significant predictor for adverse outcomes after bypass surgery. However, the mortality rate was not significantly higher in the obese group compared to the non-obese group [25]. Likewise, all-cause in-hospital mortality was not significantly higher (*p* = 0.651) in obese patients compared to normal-weight patients in our study.

Obese patients in our study were not younger compared to non-obese patients, but they had a higher prevalence of comorbidities such as diabetes mellitus (*p* < 0.001), COPD (*p* = 0.002), chronic renal insufficiency (*p* = 0.007), and dialysis (*p* = 0.006). Other illnesses such as history of arterial hypertension, hyperlipidaemia, pulmonary hypertension, and peripheral vascular disease were similar in both groups. However, the short-term follow-up might not show the negative impact of obesity on mortality, and it may lead to potential bias [26,27].

The effects of obesity on wound healing are well-known [28,29]. Note that the authors all found that obese patients are at higher risk of deep sternal wound infections after open heart surgery [29]. Furthermore, inadequate haemostasis and the extreme use of diathermy were reported to increase the postoperative proliferation of microorganisms within the wound [28,29]. In addition, prolonged operative time and insufficient use of antibiotics might relieve bacterial contamination and lead to faster development of deep sternal wound infections in obese patients [28,30]. However, in our analysis, we found a significantly higher (*p* = 0.014) wound infection rate in non-obese patients compared to obese patients. Note that this may be due to the fact that both internal mammary arteries were used more frequently in the non-obese group. Several studies reported the association between the use of both internal thoracic artery (ITA) grafts and the increased risk of wound infection after bypass surgery [31,32]. These authors suggested that the removal of the two mentioned grafts may lead to decreased blood supply to the tissue, which is associated with an increased wound infection rate postoperatively [30,31,32].

The appropriate timing for surgical treatment in patients with NSTEMI and STEMI has been the subject of critical debate in recent years [33,34]. The recent guidelines have not provided any precise recommendations regarding the right operative timing for the above mentioned patients’ cohort [35]. Hochman et al. [36] showed that early (<72 h) CABG procedures in patients after STEMI in cardiogenic shock was associated with improved outcomes. The right timing for surgical therapy in patients with NSTEMI in cardiogenic shock was critically discussed [35]. Moreover, several studies stated that patients in acute coronary syndrome (ACS) who underwent CABG in the first 72 h showed adverse outcomes and higher mortality postoperatively [37,38]. However, patients were not divided into ACS subgroups, which could affect results [38]. Liakopoulos et al. [33] reported evidence of a lower survival rate associated with the strategy of emergency revascularisation in patients with ACS and a risk factor for in-hospital mortality. In contrast, Davierwala et al. [39] did not show any statistical difference regarding in-hospital mortality between emergency and delayed surgery in patients after NSTEMI only. Further authors hypothesized that delaying bypass procedures in patients after NSTEMI does not improve outcomes and could be associated with immense resource use and costs [40]. The short-, mid-, and long-term outcomes after STEMI compared to NSTEMI in obese patients after CABG surgery are controversial [33,34,40]. A previously published study showed that patients with STEMI showed poorer outcomes in the long-term follow-up compared to patients with NSTEMI after bypass surgery [41]. A significantly higher mortality rate was also described in patients with STEMI compared to patients with NSTEMI after acute myocardial infarction [41]. Additionally, a STEMI was shown to be an independent predictor of higher mortality [33,41]. We are able to demonstrate that STEMI still remains a relevant predictor of in-hospital mortality in our study.

Several studies mentioned that obesity might be a risk factor for ischemic stroke [42,43]. In contrast, further authors found better functional outcomes after stroke in obese patients compared to non-obese patients after bypass surgery [44]. It could be hypothesized that the inflammatory response could be reduced due to the “protective” role of peripheral fat in overweight patients [44]. This controversial finding was described as the “obesity paradox” in previous studies [42,43,44,45]. We did not detect statistically significant differences in transient ischaemic attacks (TIA) and strokes in the obese group compared to the non-obese group in our study group. However, after dividing the obese patients by obesity classes, we found a significantly higher (*p* = 0.008) rate of TIA in patients from obesity class II compared to other obesity classes.

As previously described, obesity was associated with an increased risk of renal failure and the development of end-stage renal disease after cardiac surgery [46,47]. Authors mentioned that oxidative stress and endothelial dysfunction could play a crucial role in the pathogenesis of kidney failure postoperatively [46]. In addition, increased inflammatory responses and endothelial dysfunction in obese patients were further risk factors of postoperative kidney failure development [48,49]. Moreover, further epidemiologic studies found that obesity was an independent predictor of chronic renal insufficiency after open-heart surgery [46,50]. The number of dialyses was also significantly higher in obese patients compared to non-obese patients after OPCAB procedures in our study (*p* = 0.019). In addition, patients belonging to obesity class II were statistically significantly more likely to have dialysis (<0.001) than patients belonging to classes I and III. These results could be replicated both before and after PSM analysis. According to our results, 28.6% of patients from obesity class II had dialysis before PSM, and 20.8% had dialysis after PSM. In contrast, Moulton et al. [51] could not find any differences regarding end-stage renal disease between these groups in their analysis [51]. The controversial results from the already mentioned trials could be related to the small patient cohorts and could be potentially biased [46,50,51]. Therefore, larger, ideally prospective trials are needed to verify or controvert these findings in the future.

In conclusion, the expertise of the heart centre and perioperative treatment might have a significant influence on the patient’s outcome after bypass surgery, and they should be taken into consideration in our outcome evaluation.

## 5. Study Limitations

Several limitations are associated with this study. First, it was a retrospective analysis at two centres with a non-large patient cohort. The sample size was not calculated due to the investigative character of the study. In addition, we focused on short-term outcomes and did not investigate long-term outcomes. Additionally, OPCAB surgery was performed by different surgeons, which could lead to a possible bias in the results presented.

## 6. Conclusions

Obesity was not associated with increased in-hospital mortality in patients undergoing OPCAB procedures in our study. Nevertheless, STEMI and reoperation rates were relevant predictors of in-hospital mortality. The results show that obesity did not significantly affect the risk of secondary endpoints, except dialysis, during the short follow-up period. Thus, OPCAB surgery remains a very safe procedure even for obese patients.

## Figures and Tables

**Table 1:** Patients’ baseline preoperative demographics.

	Before PSM *n* = 332			After PSM *n* = 248		
	Non-Obese (*n* = 193)	Obese (*n* = 139)	*p*-Value	Non-Obese (*n* = 124)	Obese (*n* = 124)	*p*-Value
Age (years), (min/max)	67(39/88)	67(44/86)	0.451	68(39/88)	66(44/86)	0.106
Female gender, *n* (%)	37 (19.2%)	48 (34.5%)	0.002	31 (25.0%)	39 (31.5%)	0.259
Euroscore II (%), mean ± SD	4.0 ± 2.6	4.2 ± 2.1	0.232	4.3 ± 2.9	4.0 ± 1.9	0.178
LV-EF (%), mean ± SD	48 ± 13	47 ± 14	0.354	47 ± 13	48 ± 14	0.281
LM CAD, *n* (%)	91 (48.1%)	42 (30.9%)	0.002	50 (40.3%)	40 (32.3%)	0.187
History of NSTEMI, *n* (%)	59 (30.7%)	49 (35.5%)	0.362	39 (31.5%)	39 (31.5%)	1.000
History of STEMI, *n* (%)	24 (12.6%)	22 (15.9%)	0.383	16 (12.9%)	21 (16.9%)	0.373
Cardiogenic shock, *n* (%)	15 (7.8%)	21 (15.2%)	0.033	13 (10.5%)	19 (15.3%)	0.256
Previous PTCA, *n* (%)	56 (29.2%)	44 (32.1%)	0.566	32 (25.8%)	39 (31.5%)	0.325
Previous stroke, *n* (%)	9 (4.7%)	10 (7.3%)	0.317	7 (5.6%)	8 (6.5%)	0.790
Urgent procedure, *n* (%)	15 (7.8%)	21 (15.2%)	0.033	13 (10.5%)	19 (15.3%)	0.256
Reoperation, *n* (%)	17 (8.9%)	11 (8.2%)	0.838	9 (7.3%)	10 (8.3%)	0.768
DM, *n* (%)	59 (30.7%)	71 (51.1%)	<0.001	50 (40.3%)	60 (48.4%)	0.201
Insulin-dependent DM (%)	20 (10.4%)	13 (9.4%)	0.766	17 (13.7%)	10 (8.1%)	0.160
HbA1C (%), (min/max)	6.4(5.1/12.6)	6.5(5.0/9.6)	0.356	6.6(5.1/12.6)	6.5(5.0/9.6)	0.272
Hyperlipidaemia, *n* (%)	178 (92.7%)	123 (88.5%)	0.187	114 (91.9%)	113 (91.1%)	0.820
PVD, *n* (%)	39 (20.3%)	33 (23.4%)	0.394	31 (25.0%)	29 (23.4%)	0.767
Arterial hypertension, *n* (%)	184 (95.8%)	133 (95.7%)	0.947	120 (96.8%)	120 (96.8%)	1.000
PH, *n* (%)	5 (2.6%)	7 (5.0%)	0.247	1 (0.8%)	4 (3.2%)	0.370
COPD, *n* (%)	20 (10.4%)	32 (23.4%)	0.002	18 (14.5%)	27 (21.8%)	0.138
Dialysis, *n* (%)	2 (1.0%)	9 (6.6%)	0.006	2 (1.6%)	5 (4.0%)	0.446
CKD, *n* (%)	16 (8.3%)	25 (18.2%)	0.007	16 (12.9%)	19 (15.3%)	0.584

LV-EF, left ventricular ejection fraction; PVD, peripheral vascular disease; COPD, chronic obstructive pulmonary disease; STEMI, ST-elevation myocardial infarction; NSTEMI, non-ST-elevation myocardial infarction; PH; pulmonary hypertension; DM, diabetes mellitus; LM, left main; CAD, coronary artery disease; CKD, chronic kidney disease; PSM, propensity score matching.

**Table 2:** Intraoperative data.

	Before PSM *n* = 332			After PSM *n* = 248		
	Non-Obese (*n* = 193)	Obese (*n* = 139)	*p*-Value	Non-Obese (*n* = 124)	Obese (*n* = 124)	*p*-Value
Use of two ITA grafts, *n* (%)	110 (57.9%)	68 (50.0%)	0.158	62 (50.4%)	61 (49.2%)	0.849
TAR, *n* (%)	105 (55.3%)	53 (39.5%)	0.005	63 (51.2%)	49 (40.2%)	0.082
T-graft technique, *n* (%)	106 (55.8%)	48 (35.8%)	<0.001	62 (50.4%)	46 (37.7%)	0.045
Endoscopic SVG, *n* (%)	52 (27.5%)	49 (36.0%)	0.102	42 (34.1%)	43 (34.7%)	0.930
Heartstring, *n* (%)	64 (34.0%)	57 (41.9%)	0.148	49 (40.5%)	52 (41.9%)	0.819
Catecholamine use, *n* (%)	185 (99.1%)	133 (96.4%)	0.204	117 (98.3%)	120 (96.8%)	0.442
Temporary pacer, *n* (%)	108 (56.8%)	81 (60.0%)	0.570	70 (56.9%)	73 (59.8%)	0.642
ECMO intraoperative, *n* (%)	0 (0.0%)	2 (1.4%)	0.174	0 (0.0%)	2 (1.6%)	0.498
IABP intraoperative, *n* (%)	5 (2.6%)	2 (1.5%)	0.704	3 (2.4%)	2 (1.6%)	0.658
OP time, (min/max)	169 (108/370)	179 (80/330)	0.974	169 (108/370)	179 (80/330)	0.865

ECMO, extracorporeal membrane oxygenation; IABP, intra-aortic balloon pump; ITA, internal thoracic artery; SVG, saphenous vein grafts; TAR, totally arterial revascularization; OP time, operation time.

**Table 3:** Postoperative data.

	Before PSM *n* = 332			After PSM *n* = 248		
	Non-Obese (*n* = 193)	Obese (*n* = 139)	*p*-Value	Non-Obese (*n* = 124)	Obese (*n* = 124)	*p*-Value
TIA, *n* (%)	3 (1.6%)	10 (7.2%)	0.009	2 (1.6%)	8 (6.5%)	0.102
Stroke, *n* (%)	1 (0.5%)	0 (0.0%)	0.395	0 (0.0%)	0 (0.0%)	1.000
Delirium, *n* (%)	17 (8.9%)	20 (14.5%)	0.113	13 (10.6%)	17 (13.7%)	0.450
LCOS after surgery, *n* (%)	5 (2.6%)	7 (5.1%)	0.249	2 (1.7%)	6 (4.8%)	0.281
CK, 48 h, U/L, mean ± SD	635 ± 752	860 ± 1316	0.086	681 ± 761	909 ± 1361	0.162
CK-MB, 48 h, U/L, mean ± SD	39 ± 137	24 ± 20	0.256	46 ± 171	24 ± 21	0.215
Lactate 48 h, mmol/L, mean ± SD	1.5 ± 1.2	1.5 ± 1.1	0.785	1.5 ± 0.8	1.4 ± 0.7	0.516
Creatinine 48 h, mg/dL, mean ± SD	1.0 ± 0.4	1.4 ± 2.8	0.122	1.0 ± 0.3	1.4 ± 2.9	0.211
Acute kidney failure, *n* (%)	11 (5.8%)	11 (8.0%)	0.436	8 (6.6%)	7 (5.6%)	0.765
Dialysis, *n* (%)	1 (0.5%)	14 (10.1%)	<0.001	1 (0.8%)	9 (7.3%)	0.019
Wound infection, *n* (%)	23 (12.0%)	8 (5.8%)	0.058	14 (11.3%)	4 (3.2%)	0.014
Plastic covering, *n* (%)	4 (2.1%)	1 (0.7%)	0.405	3 (2.4%)	1 (0.8%)	0.622
PPI, *n* (%)	4 (2.1%)	2 (1.4%)	0.662	4 (3.3%)	1 (0.8%)	0.211
Bleeding with reoperation, *n* (%)	10 (5.2%)	3 (2.2%)	0.162	8 (6.5%)	3 (2.4%)	0.216
ICU stay, days, median(min/max)	2 (1/3)	2(1/6)	0.147	2 (1/3)	2 (1/5)	0.314
Hospital stay, days, median(min/max)	9 (5/18)	9 (5/20)	0.195	9 (5/17)	9 (5/19)	0.058
In-hospital mortality, *n* (%)	3 (1.6%)	5 (3.6%)	0.286	2 (1.6%)	3 (2.4%)	0.651

TIA, transient ischemic attack, LCOS; low cardiac output syndrome; PPI, permanent pacemaker implantation; ICU, intensive care unit.

**Table 4:** Distribution of overweightness due to obesity classes (I, II, and III).

	Before PSM Obesity Class (*n* = 139)				After PSM Obesity Class (124)			
	I (*n* = 96)	II (*n* = 29)	III (*n* =1 4)	*p*-Value	I (*n* = 86)	II (*n* = 24)	III (*n* = 14)	*p*-Value
TIA, *n* (%)	7 (7.3%)	0 (0.0%)	3 (21.4%)	0.002	5 (5.8%)	0 (0.0%)	3 (21.4%)	0.008
Stroke, *n* (%)	0 (0.0%)	0 (0.0%)	0 (0.0%)	1.000	0 (0.0%)	0 (0.0%)	0 (0.0%)	1.000
Delirium, *n* (%)	12 (12.5%)	5 (17.9%)	3 (21.4%)	0.414	9 (10.5%)	5 (20.8%)	3 (21.4%)	0.475
LCOS after surgery, *n* (%)	4 (4.2%)	3 (10.7%)	0 (0.0%)	0.234	4 (4.7%)	2 (8.3%)	0 (0.0%)	0.444
Acute kidney failure, *n* (%)	6 (6.3%)	5 (17.9%)	0 (0.0%)	0.133	3 (3.5%)	4 (16.7%)	0 (0.0%)	0.063
Dialysis, *n* (%)	5 (5.2%)	8 (28.6%)	1 (7.1%)	<0.001	3 (3.5%)	5 (20.8%)	1 (7.1%)	<0.001
Wound infection, *n* (%)	7 (7.3%)	1 (3.6%)	0 (0.0%)	0.165	3 (3.5%)	1 (4.2%)	0 (0.0%)	0.369
Plastic covering, *n* (%)	1 (1.0%)	0 (0.0%)	0 (0.0%)	0.790	1 (1.2%)	0 (0.0%)	0 (0.0%)	0.847
PPI, *n* (%)	1 (1.0%)	1 (3.6%)	0 (0.0%)	0.399	1 (1.2%)	0 (0.0%)	0 (0.0%)	0.195
Bleeding with reoperation, *n* (%)	1 (1.0%)	1 (3.6%)	1 (7.1%)	0.172	1 (1.2%)	1 (4.2%)	1 (7.1%)	0.113
In-hospital mortality, *n* (%)	2 (2.1%)	2 (7.1%)	1 (7.1%)	0.105	1 (1.2%)	1 (4.2%)	1 (7.1%)	0.230

LCOS, low cardiac output syndrome; TIA, transient ischemic attack; PPI, permanent pacemaker implantation.

**Table 5:** Univariate and multivariate logistic regression models of hospital mortality.

Combined Risk Factors	Univariate Logistic Regression Model		Multivariate Logistic Regression Model	
	OR (CI 95%)	*p*-Value	OR (CI 95%)	*p*-Value
Age	1.037 (0.949–1.133)	0.426	1.037 (0.957–1.123)	0.372
BMI	1.005 (0.925–1.092)	0.907	1.011 (0.961–1.062)	0.681
DM	4.308 (0.779–23.815)	0.094	4.629 (0.924–23.381)	0.062
STEMI	7.909 (1.540–40.611)	0.013	6.239 (1.507–25.823)	0.012
NSTEMI	3.745 (0.718–19.518)	0.117	2.064 (0.507–8.410)	0.312
Reoperation	6.718 (1.324–34.084)	0.022	7.320 (1.653–32.424)	0.009

STEMI, ST-elevation myocardial infarction; NSTEMI, non-ST-elevation myocardial infarction; BMI, body mass index; DM, diabetes mellitus.

## Data Availability

Data can be obtained by special request to the corresponding author.
